# Telomere Length, Oxidative Stress, and Kidney Damage Biomarkers in Fabry Nephropathy

**DOI:** 10.3390/cells14030218

**Published:** 2025-02-04

**Authors:** Tina Levstek, Erazem Bahčič, Bojan Vujkovac, Andreja Cokan Vujkovac, Tine Tesovnik, Žiga Iztok Remec, Vanja Čuk, Katarina Trebušak Podkrajšek

**Affiliations:** 1 Institute of Biochemistry and Molecular Genetics, Faculty of Medicine, University of Ljubljana, 1000 Ljubljana, Slovenia; 2 Clinical Institute for Special Laboratory Diagnostics, University Children’s Hospital, University Medical Centre Ljubljana, 1000 Ljubljana, Slovenia; 3 Centre for Fabry Disease, General Hospital Slovenj Gradec, 2380 Slovenj Gradec, Slovenia

**Keywords:** Fabry disease, nephropathy, telomere length, oxidative stress, kidney damage, biomarker, aging

## Abstract

Fabry nephropathy is a life-threatening complication of Fabry disease characterized by complex and incompletely understood pathophysiological processes possibly linked to premature aging. We aimed to investigate leukocyte telomere length (LTL), oxidative stress, and kidney damage biomarkers in relation to kidney function. The study included 35 Fabry patients and 35 age and sex-matched control subjects. Based on the estimated slope of the glomerular filtration rate, the patients were divided into two groups. Relative LTL was quantified by qPCR, urinary biomarkers 8-hydroxy-2′-deoxyguanosine (8-OHdG) and malondialdehyde (MDA) by UHPLC-MS/MS, and kidney damage biomarkers by flow cytometry. There was no statistically significant difference in LTL between Fabry patients and controls. However, a significant difference was observed in male patients compared to their matched control subjects (*p* = 0.013). Oxidative stress biomarkers showed no differences between patients and controls, while significant differences were observed in urinary IGFBP7, EGF, and OPN levels between Fabry patients with stable kidney function and those with progressive nephropathy (FDR = 0.021, 0.002, and 0.013, respectively). Significant differences were also observed in plasma levels of cystatin C, TFF3, and uromodulin between patients with progressive nephropathy and controls (all FDR = 0.039). Along with these biomarkers (FDR = 0.007, 0.017, and 0.010, respectively), NGAL also exhibited a significant difference between the two patient groups (FDR = 0.017). This study indicates accelerated telomere attrition, which may be related to disease burden in males. Furthermore, analyses of urinary oxidative stress markers revealed no notable disparities between the different kidney function groups, indicating their limited utility. However, promising differences were found in some biomarkers of kidney damage in urine and plasma.

## 1. Introduction

Fabry disease (FD) is a lysosomal storage disorder resulting from disease-causing variants in the *GLA* gene [1]. It is characterized by the reduced activity of α-galactosidase A, leading to the accumulation of glycosphingolipids, particularly globotriaosylceramide (Gb3) and its deacetylated form, globotriaosylsphingosine (lyso-Gb3) in various cell types throughout the body [2,3,4]. Progressive organ damage, including heart disease, cerebrovascular complications, and nephropathy, underlies the premature morbidity and mortality observed in Fabry patients [5,6,7]. Before the introduction of enzyme replacement therapy (ERT), Fabry patients had a significantly lower life expectancy than the general population. Data from the Fabry Registry show that untreated males have a life expectancy shortened by more than 15 years and females by almost 5 years [8], emphasizing the association of FD with premature aging [9]. At the molecular and cellular level, several features are associated with the aging process, namely, genomic instability, telomere shortening, altered intracellular communication, epigenetic changes, impaired proteostasis, deregulated nutrient sensing, mitochondrial dysfunction, cellular senescence, and stem cell exhaustion [10]. The deregulation of most of these processes has also been demonstrated in FD [11].

Telomeres, the protective caps at the ends of chromosomes, are widely recognized as markers of biological aging and play a central role in cellular senescence and organismal aging [12]. Accelerated telomere attrition has been reported in several age-related disorders [13,14,15]. The role of telomere attrition and kidney function is not completely understood, as the reported results are inconclusive [13]. In FD, the accumulation of Gb3 and lyso-Gb3 has been found to progress in all kidney cells [16,17] and is followed by the dysregulation of several cellular processes that disrupt cellular homeostasis [18,19]. The combined effect of Gb3 accumulation and the activation of pathogenic cascades leads, among others, to the activation of an inflammatory response [20] and increased oxidative stress. The exposure of cultured endothelial cells to Gb3 has been shown to increase oxidative stress, as evidenced by increased levels of reactive oxygen species and the upregulated expression of cell adhesion molecules [21]. Additionally, proteomics studies have shown increased levels of inflammatory markers in urine [22]. Both inflammation and oxidative stress contribute to increased telomere attrition [23,24], as telomeres are susceptible to oxidation due to their high guanine abundance [25].

While albuminuria and proteinuria serve as conventional biomarkers of kidney function, their sensitivity in detecting the onset of nephropathy is limited [26]. Given the potential of early disease-specific therapy (DST) to attenuate the progression of kidney impairment [27], the identification of biomarkers associated with early kidney dysfunction is essential for optimizing FD management. Therefore, this study aims to evaluate the interplay of leukocyte TL (LTL), oxidative stress, and biomarkers of kidney damage in relation to kidney function in Fabry patients. We aimed to investigate the underlying pathophysiological mechanisms of Fabry nephropathy and to identify potential biomarkers for early diagnosis or prognosis of disease progression.

## 2. Materials and Methods

### 2.1. Study Participants

A total of 35 Fabry patients were recruited from the Slovenian National Centre for Fabry Disease at the General Hospital Slovenj Gradec. The inclusion criteria required participants to be over 18 years old, have genetically confirmed FD, and have attended at least three follow-ups in the last 5 years. Exclusion criteria included additional glomerular disease, uncontrolled hypertension, dialysis, pregnancy at the time of enrollment or within the past 12 months, physical or psychological disease that could interfere with the normal conduct of the study, and current or recent alcohol or drug abuse. Their *GLA* variants are listed in Table S1. All patients have the classic phenotype. Patients were divided into two cohorts: (1) Fabry patients with stable kidney function defined by a decline in the estimated glomerular filtration rate (eGFR) slope of less than 3 mL/min/1.73 m^2^/year and (2) Fabry patients with progressive nephropathy, characterized by a decline in eGFR slope exceeding 3 mL/min/1.73 m^2^/year [28]. Kidney function was assessed using the Chronic Kidney Disease Epidemiology Collaboration (CKD-EPI) 2009 equation [29].

In addition, 35 age- and sex-matched control subjects were included. They were defined by the absence of kidney disease or systemic disease that could cause nephropathy (i.e., diabetes, uncontrolled arterial hypertension), a normal age-adjusted eGFR, and no albuminuria or proteinuria, as indicated by a urinary albumin-to-creatinine ratio (UACR) of ≤3 g/mol and a urinary protein-to-creatinine ratio (UPCR) of ≤20 g/mol.

The study protocol was approved by the Slovenian Ethics Committee for Research in Medicine (approval numbers: 0120-260/2020/6, 0120-521/2020/3, and 0120-260/2020/12). All participants provided written informed consent in accordance with the Declaration of Helsinki.

### 2.2. Sample Collection and Processing

Urine and blood samples were collected during the annual follow-up examinations. Urine samples were centrifuged within 3 h at 2000× *g* for 15 min to remove cells and debris. The supernatant was then transferred to new tubes and stored at −80 °C. Blood samples were collected in EDTA tubes and centrifuged at 2000× *g* for 10 min. Plasma was then removed and centrifuged again at 2000× *g* for 5 min to deplete thrombocytes. The resulting plasma was aliquoted and stored at −80 °C. The cells used for DNA isolation were frozen at −20 °C. The control subjects provided blood and urine samples according to the same protocols.

### 2.3. Assessment of Relative Leukocyte Telomere Length by Quantitative PCR

The FlexiGene DNA Kit (Qiagen, Hilden, Germany) was used to extract genomic DNA from frozen buffy coats following the manufacturer’s protocol. The quality and quantity of DNA samples were determined spectrophotometrically using the NanoDrop One (Thermo Fisher Scientific, Waltham, MA, USA). The isolated DNA was stored at 4 °C. Monochrome multiplex quantitative polymerase chain reaction (mmqPCR) was used to measure relative LTL, following the previously described protocol [30,31].

### 2.4. Measurement of Oxidative Stress Biomarkers in Urine Using UHPLC-MS/MS

The method was developed on the basis of a previously published article [32]. Stock solutions of 1 mg/mL were prepared for both analytes. A malondialdehyde (MDA) solution was prepared in methanol and stored in glass vials filled with nitrogen gas. Then, 8-hydroxy-2′-deoxyguanosine (8-OHdG) was diluted in HPLC-grade water (both Cayman Europe, Tallinn, Estonia). The initial 8-OHdG working solution for the standard curve had a concentration of 1 µg/mL, and the initial MDA working solution had a concentration of 10 µg/mL. Both were prepared in ethanol. A 5-point standard curve was obtained using working solutions of MDA and 8-OHdG. The final concentrations of the standard curve were 20, 15, 10, 5, and 0.5 ng/mL for both analytes.

The derivatization reagent 2,4-dinitrophenylhydrazine (DNPH) was prepared in a mixture of water, acetonitrile (ACN), and acetic acid (8:1:1, *v*/*v*) at a concentration of 0.05 M. To extract the DNPH solution, it was vortexed twice with 5 mL of *n*-hexane for 5 min. The organic layer was then discarded, and the DNPH was filtered through a 0.2 µm filter (Sartorius AG, Goettingen, Germany) and stored at 4 °C. For the derivatization of MDA, 200 µL of DNPH was added to 0.5 mL of blank, standard, or urine sample, followed by 20 µL of an internal standard mixture. The internal standard mixture contained d_2_-MDA and ^15^N_5_-8-OHdG at a final concentration of 5 ng/mL for each compound (Cambridge Isotope Laboratories, Inc., Tewksbury, MA, USA). Samples were analyzed in duplicate. After incubation at room temperature for 30 min, 2.1 mL of 2% formic acid was added to the derivatized sample.

Solid phase extraction was performed with EVOLUTE^®^ EXPRESS ABN (60 mg, 3 mL) cartridges (Biotage, Uppsala, Sweden). The cartridges were first conditioned with 2 mL of 100% methanol, followed by 2 mL of HPLC water. The derivatized sample was then loaded onto the cartridge and washed with 2 mL of 5% methanol. The cartridges were then vacuum-dried. The target biomarkers were eluted with 1 mL of methanol, followed by 1 mL of ethyl acetate. The eluate was evaporated with a stream of nitrogen and reconstituted with 150 µL of water/methanol (8:2 *v*/*v*) before being transferred to vials with glass inserts for ultra-high-performance liquid chromatography with tandem mass spectrometry (UHPLC-MS/MS) analysis.

Target analytes were quantified using a Waters Acquity I-class UHPLC-integrated HPLC-MS/MS system coupled to a Waters Xevo TQ-XS triple quadrupole mass spectrometer (both Waters Corporation, Milford, MA, USA). A Poroshell 120 SB-Aq 2.1 × 100 mm, 2.7 μm LC column (Agilent Technologies, Santa Clara, CA, USA) was used for the chromatographic separation of the target analytes. The mobile phases used were as previously described [32]. Then, 0.01% of acetic acid in water was labeled as A, and 0.01% of acetic acid in methanol was labeled as B. We adjusted the liquid chromatography profile to accommodate the UHPLC column. The initial mobile phase consisted of 80% A for 6 s, followed by a transition to 55% A over 39 s. Thereafter, the A composition gradually decreased to 45% until 5.1 min. At 5.6 min, the composition transitioned completely to 100% B and remained constant for 8 min. Finally, the composition returned to its initial state, with 20% A and 80% B at 8.5 min, and it maintained this ratio until the end of the 10 min run (Table S2). For quantification, we employed the multiple reaction monitoring (MRM) MS method in the positive ionization mode. The compound-specific MS/MS parameters, the *m*/*z* transitions of MRM, and the ion source parameters were optimized by injecting an individual standard solution of the analyte at 1 µg/mL.

### 2.5. Measurement of Kidney Damage Biomarkers in Urine and Plasma on a Flow Cytometer

The LEGENDplex™ Human Kidney Function Panel 1 Mix and Match SubPanel and the LEGENDplex™ Human Kidney Function Panel 2 Mix and Match SubPanel (BioLegend, San Diego, CA, USA) were utilized to measure kidney damage biomarkers in urine and plasma samples, respectively. These are multiplex bead-based assay panels that employ the same principles as sandwich immunoassays. Panel 1 simultaneously quantified nine proteins in urine, including insulin-like growth factor binding protein 7 (IGFBP7), β2-microglobulin (B2MG), cystatin C, clusterin (ApoJ), epidermal growth factor (EGF), albumin, OPN (osteopontin), TFF3 (trefoil factor 3), and uromodulin. Panel 2 quantified seven human proteins in plasma samples, including IGFBP7, neutrophil gelatinase-associated lipocalin (NGAL), cystatin C, tissue inhibitor of metalloproteinases 2 (TIMP2), OPN, TFF3, and uromodulin. The concentration of each analyte was determined using standard curves. The assays were performed on a filter plate following the manufacturer’s protocol. All samples were measured in duplicate using a BD FACSLyric™ System flow cytometer (Becton, Dickinson and Company, NJ, USA). A minimum of 5000 beads were analyzed for each sample. The FCS files were analyzed using BioLegend’s LEGENDplex™ (https://www.biolegend.com) data analysis software.

### 2.6. Statistical Analysis

Statistical analyses were performed with RStudio version 4.3.0 (R Core Team, Vienna, Austria). Descriptive statistics were used to summarize the characteristics of the study population. The Shapiro–Wilk test was used to test normality. Data were expressed as medians (25–75%) for continuous variables and as frequencies (percentage) for categorical variables. Continuous variables were compared between groups using the Mann–Whitney U test. Linear regression and ANCOVA were used to adjust for potential confounders and assess the differences between groups while controlling for covariates. Spearman’s Rho coefficient was used to assess the correlation between continuous variables. A *p* value of <0.05 was considered significant. When indicated, correction for multiple testing was performed using false discovery rates (FDRs). A total of 70 patients were enrolled, which exceeded the calculated sample size of 67. The sample size was calculated using simulation-based methods and the pwr package, assuming a medium effect size (d = 0.5), a test power of 80%, and an alpha level of 0.05.

## 3. Results

### 3.1. Participant Characteristics

The study included 35 patients with genetically confirmed FD and an equal number of age- and sex-matched control subjects. In patients with stable kidney function, daily proteinuria was 0.14 (0.09–0.28) g/day, with an eGFR slope of −1.0 (−1.8–−0.5) mL/min/1.73 m^2^/year. Significant differences in UPCR and serum creatinine were observed between the patients with stable kidney function and matched control subjects. This finding indicates that proteinuria is an early sign of Fabry nephropathy, present already in patients with stable kidney function. Similarly, patients with progressive nephropathy showed statistically significant differences in eGFR, UPCR, and serum creatinine levels compared to control subjects. Specifically, patients with progressive nephropathy had daily proteinuria of 0.39 (0.18–1.23) g/day, in addition to an eGFR slope of −4.1 (−5.5–−3.9) mL/min/1.73 m^2^/year. The detailed data are shown in Table 1.

A total of 17 (48.6%) patients received ERT with a median duration of 12.6 (9.0–14.5) years. Of these, 8 (22.9%) were males with a median duration of 13.6 (11.0–15.3) years, while the median duration for females was 9.0 (5.0–14.5) years. A total of 8 patients received agalsidase alpha, 7 received agalsidase beta, and 2 were given pegunigalsidase alpha after switching from agalsidase beta in 2018. The median duration of ERT was 12.0 (7.5–15.0) years. It is important to acknowledge that some patients declined DST for personal reasons, while others were diagnosed with FD before ERT was available. The median time from diagnosis to the start of the ERT was 1.5 (0.0–3.5) years. 

### 3.2. Telomere Length

**Figure 1:**
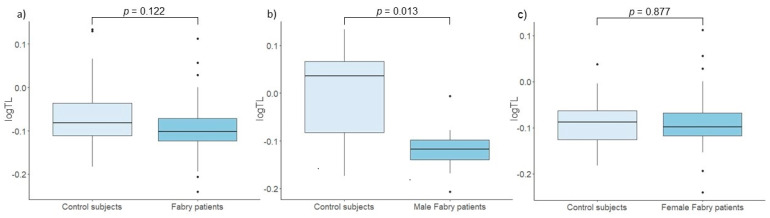
Comparison of leukocyte telomere length (TL) between (**a**) control subjects and Fabry patients, (**b**) control subjects and male Fabry patients, and (**c**) control subjects and female Fabry patients.

**Figure 2:**
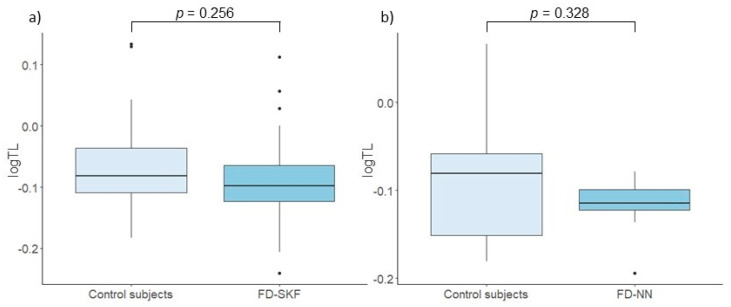
Comparison of leukocyte telomere length (TL) between (**a**) control subjects and Fabry patients with stable kidney function (FD-SKF) and (**b**) control subjects and Fabry patients with progressive nephropathy (FD-NN).

As expected, a significant negative correlation was found between LTL and age (*p* = 0.049, Rho = −0.24). No significant difference in relative LTL was observed between Fabry patients and their matched control subjects (*p* = 0.122). However, a sex-specific analysis showed a statistically significant difference between Fabry males and their control subjects (*p* = 0.013). Conversely, there was no difference in relative LTL between Fabry females and control subjects (*p* = 0.877). The results are shown in Figure 1. Furthermore, assessment of the impact of kidney function on LTL revealed no significant discrepancy between patients with stable kidney function and their controls (*p* = 0.256), nor between patients with progressive nephropathy and control subjects (*p* = 0.328) (Figure 2). Furthermore, linear regression analysis, adjusted for age and DST, showed no statistically significant association between LTL and nephropathy status (*p* = 0.605). There were also no significant correlations observed between LTL and eGFR (*p* = 0.683, Rho = −0.07) or UACR (*p* = 0.888, Rho = 0.03) in Fabry patients.

#### Oxidative Stress Biomarkers in Urine

**Figure 3:**
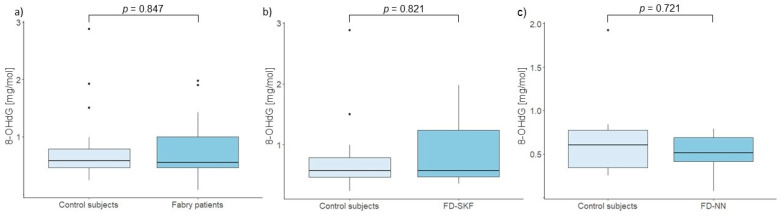
Comparison of urinary 8-OHdG levels between (**a**) control subjects and Fabry patients, (**b**) control subjects and Fabry patients with stable kidney function (FD-SKF), and (**c**) control subjects and Fabry patients with progressive nephropathy (FD-NN).

Our study revealed no significant differences in 8-OHdG levels between Fabry patients with progressive nephropathy and matched control subjects (*p* = 0.721). Similarly, no difference was found between patients with stable kidney function and control subjects (*p* = 0.821). There was also no difference between Fabry patients and control subjects when comparing 8-OHdG levels (*p* = 0.847). The results are shown in Figure 3. Even after adjustment for DST, no significant difference was observed between patients with stable kidney function and those with progressive nephropathy (*p* = 0.076).

**Figure 4:**
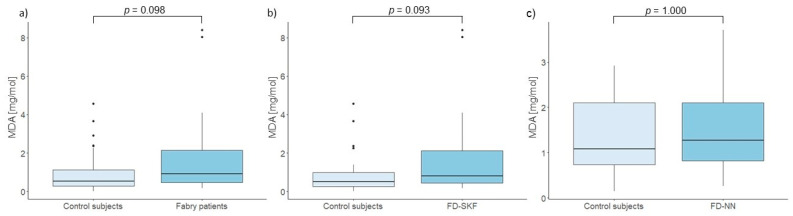
Comparison of urinary MDA levels between (**a**) control subjects and Fabry patients, (**b**) control subjects and Fabry patients with stable kidney function (FD-SKF), and (**c**) control subjects and Fabry patients with progressive nephropathy (FD-NN).

Our results also showed no significant differences in urinary MDA levels between Fabry patients and control subjects (*p* = 0.098). Similarly, no significant differences were found when comparing MDA levels between Fabry patients with stable kidney function and control subjects (*p* = 0.093) and between Fabry patients with progressive nephropathy and matched control subjects (*p* = 1.000). These results are presented in Figure 4. Even after adjustment for DST, no significant difference in MDA was observed between patients with stable kidney function and those with progressive nephropathy (*p* = 0.774).

**Figure 5:**
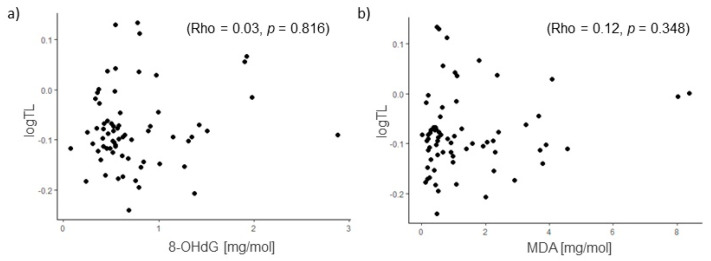
Correlation between urinary concentration of (**a**) 8-OHdG or (**b**) MDA and relative leukocyte telomere length (TL).

Furthermore, no significant correlation was found between 8-OHdG levels and relative LTLs (Rho = 0.03, *p* = 0.816), nor between MDA levels and relative LTLs (Rho = 0.12, *p* = 0.348) (Figure 5). No sex differences in urinary levels of 8-OHdG and MDA were observed either.

### 3.3. Urinary Biomarkers of Kidney Damage

When patients with stable kidney function or patients with progressive nephropathy were compared with the corresponding control subjects, a statistically significant difference was found in the concentration of albumin in urine (FDR = 0.038 in both cases), as shown in Table 2. Additionally, statistically significant differences were found in the urine concentration of IGFBP7, EGF, and OPN (FDR = 0.021, 0.002, and 0.013, respectively) between patients with stable kidney function and those with progressive nephropathy. After adjustment for DST, EGF and albumin levels differed significantly (FDR = 0.027 and 0.007, respectively) between the patient groups (Table S3). All parameters measured in urine were normalized to urinary creatinine levels. Most samples had concentrations of cystatin C and clusterin below the limit of quantification and were therefore excluded from statistical analysis. No significant sex-specific differences were observed in urinary kidney damage biomarkers.

### 3.4. Plasma Biomarkers of Kidney Damage

Table 3 shows the plasma concentration of seven biomarkers. A comparison between patients with progressive nephropathy and control subjects revealed statistically significant differences in the concentration of cystatin C, TFF3, and uromodulin (FDR = 0.039 for all). In addition, significant differences in the plasma levels of NGAL, cystatin C, TFF3, and uromodulin were observed between patients with stable kidney function and those with progressive nephropathy (FDR = 0.017, 0.007, 0.017, and 0.010, respectively). However, after adjustment for DST, a significant difference was found for NGAL and cystatin C (FDR = 0.017 for both) (Table S4). However, no sex-specific or differences in plasma levels of kidney damage biomarkers were found between patients with stable kidney function and their control subjects.

## 4. Discussion

Given the shorter lifespan of Fabry patients compared to the general population, we hypothesized that disease burden may accelerate telomere shortening in Fabry patients. While our study found no significant difference in relative LTL between Fabry patients and control subjects overall, notable gender differences were uncovered. Specifically, male Fabry patients had significantly shorter LTL compared to age-matched control subjects, a discrepancy that was not observed in female Fabry patients and their control subjects. Although FD is inherited X-linked, female patients may also exhibit symptoms ranging from asymptomatic to severe phenotypes resembling the classic male clinical picture [5]. Our patient cohort had a higher disease burden in males, which likely contributes to the observed LTL discrepancy. These findings are consistent with a previous study that confirmed a significant discrepancy in LTL between male Fabry patients and control subjects [9]. In particular, an assessment of telomerase activity in the same study revealed intriguing results; telomerase activity was significantly increased in female patients compared to control subjects, whereas no significant difference was found between male patients and control subjects [9]. This difference in telomerase activity could possibly explain the lack of observed differences between female Fabry patients and control subjects.

As Fabry nephropathy contributes significantly to overall disease morbidity and mortality, we also wanted to investigate the association between LTL and kidney function. Indeed, several studies have already assessed the relationship between TL and kidney function in various chronic kidney diseases (CKDs), but the results are ambiguous [13]. Most studies in this area have focused on the assessment of relative LTL, which has been found to correlate positively with TL in the kidney cortex [33]. In our study, we found no discernible difference in relative LTL between patients with stable kidney function and those with progressive nephropathy. This result is consistent with previous studies in Fabry patients, which also found no correlation between LTL and eGFR or the presence of proteinuria [9]. Given that the kidneys are recognized as highly sensitive to aging [34] and that the mortality rate in dialysis patients far exceeds that of the general population [35], these results are rather unexpected.

The results of our study indicated a significant negative correlation between age and relative LTL, which is reflective of the effects of repeated cell replication. Furthermore, the rate of TL attrition may also be influenced by other factors, such as oxidative stress. In FD, oxidative stress has been proposed to be involved in the progression of organ damage, with the primary accumulation products, Gb3 and lyso-Gb3, shown to induce a pro-oxidant state [36,37,38,39]. However, we found no correlation between urinary levels of 8-OHdG and MDA and relative LTL. Additionally, no statistically significant differences were observed in urinary oxidative stress markers between patients with stable kidney function and those with progressive nephropathy, or between Fabry patients and their control subjects. Although there is some evidence for a possible role of oxidative stress in cardiovascular-renal remodeling, particularly through plasma biomarkers [39], our study indicates that urinary 8-OHdG, a marker of DNA damage, and MDA, a marker of lipid peroxidation, remain relatively stable in the urine of Fabry patients, even in the presence of kidney damage. These findings are consistent with a recent study reporting that prolonged ERT reduces inflammatory biomarkers in males and mitigates oxidative stress-induced damage in both sexes [40]. This underscores the intricate interplay between oxidative stress and therapeutic interventions in FD.

The aging of the kidney is associated with functional changes and alterations in the levels of certain biomarkers. Nevertheless, identifying early kidney damage and predicting faster progression of nephropathy remain a challenge due to the lack of reliable biomarkers [26]. To address this issue, we evaluated biomarkers of kidney damage established in other CKDs for their potential applicability in Fabry nephropathy. Albuminuria, a widely used biomarker of kidney damage, showed significant differences in UACR between patients and control subjects. Additionally, the urinary levels of IGFBP7, EGF, and OPN showed significant differences between patients with stable kidney function and patients with progressive nephropathy. After adjustments for DST, OPN and albumin remained significantly different between the two groups. These biomarkers, which are known to be highly expressed in the kidney, have been associated with CKD, with increased urinary levels of IGFBP-7 and OPN and decreased levels of EGF [41,42,43,44]. Decreased urinary EGF correlates with tubular atrophy and interstitial fibrosis [42], while OPN promotes inflammation and fibrosis and regulates calcium and phosphate metabolism. Studies suggest that the suppression of OPN expression or activity may ameliorate kidney damage and improve kidney function [43,44]. Interestingly, in our study, patients with progressive nephropathy had lower OPN levels than patients with stable kidney function, which warrants further investigation in larger cohorts. Our study further explored the potential biomarkers of kidney damage in plasma and showed significant differences in the levels of cystatin C, TFF3, and uromodulin between patients with progressive nephropathy and their control subjects. In addition to these markers, NGAL also differed significantly between patients with progressive nephropathy and those with stable kidney function. Even after adjustments for DST, NGAL and cystatin C still differed significantly between the two groups. Cystatin C has been demonstrated to be a more accurate indicator of kidney function than serum creatinine. However, the data of cystatin C and eGFR are still controversial [45]. Due to the natural loss of nephrons with increasing age [34], the difference in cystatin C between patients with progressive nephropathy and those with stable kidney function may indicate age differences, as patients with progressive nephropathy tend to be older. Uromodulin is also a known marker for CKD. Reduced levels of urinary uromodulin indicate kidney damage or a loss of kidney function and are associated with adverse outcomes [46]. Contrarily, TFF3 levels increase with CKD progression and predict a CKD prognosis [47], as it is involved in several signaling pathways, including the migration, proliferation, apoptosis, angiogenesis, and inhibition of inflammation [47]. Similarly, NGAL serves as a marker of disease severity and clinical outcome in patients with CKD [48,49]. Dysregulated markers may be linked to chronic tubulointerstitial damage that remains unresponsive to DST due to late-stage, irreversible histopathologic lesions. As a result, these lesions do not respond to therapy, aside from potential stabilization [50]. These findings underscore the importance of comprehensive biomarker assessments in different biofluids to elucidate the complexity of kidney damage and enable tailored therapeutic interventions in FD management.

It is important to note some limitations of our study that need to be carefully considered when interpreting the results. First, our patient cohort was relatively small, reflecting the rarity of FD, and increasing the sample size proved difficult. Larger studies are needed in the future to validate the results of this preliminary study. Second, the effects of DST, particularly the effects on LTL in Fabry patients, remain unclear. As the patients in our cohort had undergone DST many years prior to enrollment in the study, we were unable to assess the direct impact of DST on LTL. Therefore, further research is needed to clarify the potential impact of DST on LTL in Fabry patients, although the late diagnosis of many patients may hinder such efforts. Additionally, there may be a notable difference between late-diagnosed patients and those identified through family screening. By including the duration of DST as a covariate, there is a risk of confounding the timing of diagnoses with the effects of the therapy itself. This could distort the results and give the impression that the therapy is less effective for certain groups, although this may only be due to the delayed intervention. In addition, our study focused on investigating the relationship between two urinary oxidative stress markers and relative LTL. We had also planned to include the measurement of four F_2_-isoprostanes, but methodological optimization revealed insufficient sensitivity. A more comprehensive assessment that includes multiple oxidative stress markers or a composite score for oxidative biomarkers could provide deeper insight into the role of oxidative stress in FD. Ideally, such assessments should include different tissue types to provide a more holistic understanding of oxidative stress in FD. In addition, a longitudinal approach could provide more insight into the pathology of the disease. However, as we did not find any studies that investigated the stability of biochemical markers during long-term storage, we did not perform them to avoid misinterpretation. However, the dynamics of LTL have been studied longitudinally and published recently [51].

## 5. Conclusions

The present study highlights significant differences in LTL between Fabry males and control subjects, indicating possible accelerated telomere attrition linked to the disease burden in males. Furthermore, an examination of urinary oxidative stress markers revealed no notable disparities across different kidney function groups, indicating the limited utility of urinary 8-OHdG and MDA as biomarkers for Fabry nephropathy. However, some differences were detected in urinary IGFBP-7, EGF, and OPN, as well as in plasma NGAL, cystatin C, TFF3, and uromodulin between patients with stable kidney function and those with progressive nephropathy. The findings of our preliminary study underscore the potential of these biomarkers to delineate signaling pathways that are specifically altered in Fabry patients, thus warranting further exploration and validation in future studies.

## Figures and Tables

**Table 1:** Cohort characteristics.

Variable	FD-SKF (n = 27)	Control Group (n = 27)	*p* Value	FD-PN (n = 8)	Control Group (n = 8)	*p* Value
Sex (females)	21 (77.8)	21 (77.8)	1.000	5 (62.5)	5 (62.5)	1.000
Age (years)	45.0 (35.4–51.9)	45.5 (34.8–52.0)	0.959	59.0 (51.7–59.9)	59.4 (52.1–60.4)	0.798
eGFR (mL/min/1.73 m^2^)	107.0 (95.0–111.5)	98.0 (90.5–107.0)	0.169	66.5 (49.0–77.8)	98.5 (92.5–99.3)	**0.031**
UPCR (g/mol)	12.2 (9.6–19.2)	5.5 (4.1–7.1)	**<0.001**	52.8 (9.0–123.4)	7.2 (6.3–7.4)	**0.014**
Serum creatinine (µmol/L)	63.0 (57.5–67.5)	69.0 (65.5–74.5)	**0.002**	81.5 (78.0–138.5)	69.5 (65.5–74.8)	**0.031**

FD-SKF, Fabry patients with stable kidney function; FD-PN, Fabry patients with progressive nephropathy; eGFR, estimated glomerular filtration rate; UPCR, urinary protein-to-creatinine ratio. Values in bold indicate statistically significant results.

**Table 2:** Comparison of kidney damage biomarkers measured in urine between patients and control subjects.

Urine Marker	FD-SKF (n = 27)	Control Group (n = 27)	FDR	FD-PN (n = 8)	Control Group (n = 8)	FDR
IGFBP7 (mg/mol)	53.05 (30.76–74.28)	53.11 (40.97–66.07)	0.600	25.18 (11.39–37.60)	38.98 (24.34–55.01)	0.557
B2MG (mg/mol)	6.56 (4.40–12.51)	4.85 (4.36–8.53)	0.564	7.09 (5.46–43.59)	5.13 (2.38–7.48)	0.410
EGF (mg/mol)	3.91 (2.73–4.71)	3.79 (2.94–4.56)	0.718	1.98 (1.26–2.32)	1.93 (1.59–2.70)	0.718
albumin (g/mol)	1.1 (0.6–2.1)	0.4 (0.2–0.6)	0.038	15.1 (1.3–31.6)	0.2 (1.3–0.4)	0.038
OPN (mg/mol)	7.78 (4.61–11.13)	6.88 (4.83–8.30)	0.445	2.35 (0.46–5.40)	7.82 (6.02–11.02)	0.262
TFF3 (mg/mol)	4.84 (2.78–11.40)	3.65 (2.83–5.20)	0.410	3.23 (2.22–14.13)	2.15 (1.54–3.97)	0.440
UMOD (g/mol)	1.7 (0.8–3.4)	0.6 (0.2–2.2)	0.113	0.9 (0.2–2.2)	0.6 (0.2–1.2)	0.439

FD-SKF, Fabry patients with stable kidney function; FD-PN, Fabry patients with progressive nephropathy; FDR, false discovery rate; IGFBP7, insulin-like growth factor binding protein 7; B2MG, β2-microglobulin; EGF, epidermal growth factor; OPN, osteopontin; TFF3, trefoil factor 3; UMOD, uromodulin.

**Table 3:** Comparison of kidney damage biomarkers measured in plasma between patients and control subjects.

Plasma Marker	FD-SKF (n = 27)	Control Group (n = 27)	FDR	FD-PN (n = 8)	Control Group (n = 8)	FDR
IGFBP7 (ng/mL)	113.5 (105.6–134.9)	116.6 (101.4–140.8)	0.709	156.9 (128.8–200.5)	100.3 (92.5–154.6)	0.125
NGAL (ng/mL)	177.2 (151.0–234.0)	212.9 (176.3–248.2)	0.283	267.6 (200.7–390.4)	168.1 (129.3–223.9)	0.110
cystatin C (µg/mL)	0.63 (0.49–0.70)	0.53 (0.47–0.63)	0.284	0.99 (0.86–1.51)	0.62 (0.54–0.66)	0.039
TIMP2 (ng/mL)	42.1 (21.6–51.5)	38.2 (22.4–52.0)	0.709	41.9 (20.1–60.0)	21.6 (15.3–28.4)	0.266
OPN (ng/mL)	4.71 (2.88–6.90)	3.44 (1.87–4.96)	0.111	5.42 (2.87–9.69)	3.33 (1.73–5.07)	0.344
TFF3 (ng/mL)	3.99 (3.21–5.92)	3.62 (2.81–4.66)	0.330	8.37 (6.01–11.72)	2.85 (2.44–3.22)	0.039
UMOD (ng/mL)	105.8 (79.0–138.9)	106.2 (76.7–116.7)	0.709	35.2 (21.9–46.3)	101.6 (95.4–112.3)	0.039

FD-SKF, Fabry patients with stable kidney function; FD-PN, Fabry patients with progressive nephropathy; FDR, false discovery rate; IGFBP7, insulin-like growth factor binding protein 7; NGAL, neutrophil gelatinase-associated lipocalin; TIMP2, tissue inhibitor of metalloproteinases 2; OPN, osteopontin; TFF3, trefoil factor 3; UMOD, uromodulin.

## Data Availability

The original contributions presented in the study are included in the article/Supplementary Materials. Further inquiries can be directed to the corresponding author.

## Supplementary Materials

The following supporting information can be downloaded at https://www.mdpi.com/article/10.3390/cells14030218/s1. Table S1. Variants in the GLA gene (reference sequence NM_000169.2) and characteristics of the included Fabry patients. Table S2. UHPLC flow profile and mobile phase ratios. Table S3. Comparison of urine kidney damage biomarkers between patients with stable kidney functions and those with progressive nephropathy adjusted for disease-specific therapy. Table S4. Comparison of plasma kidney damage biomarkers between patients with stable kidney functions and those with progressive nephropathy adjusted for disease-specific therapy.

## Abbreviations

The following abbreviations are used in this manuscript:

8-OGdG	8-hydroxy-2′-deoxyguanosine;
ACN	Acetonitrile;
B2MG	β2-microglobulin;
CKD	Chronic kidney disease;
DNPH	2,4-dinitrophenylhydrazine;
DST	Disease-specific therapy;
EGF	Epidermal growth factor;
eGFR	Estimated glomerular filtration rate;
ERT	Enzyme replacement therapy;
FD	Fabry disease;
FDR	False discovery rate;
Gb3	Globotriaosylceramide;
IGFBP7	Insulin-like growth factor binding protein 7;
LTL	Leukocyte telomere length;
lyso-Gb3	Globotriaosylsphingosine;
MDA	Malondialdehyde;
mmqPCR	Monochrome multiplex quantitative polymerase chain reaction;
MRM	Multiple reaction monitoring;
NGAL	Neutrophil gelatinase-associated lipocalin;
OPN	Osteopontin;
TIMP2	Tissue inhibitor of metalloproteinases 2;
TFF3	Trefoil factor 3;
TL	Telomere length;
UACR	Urinary albumin-to-creatinine ratio;
UHPLC-MS/MS	Ultra-high-performance liquid chromatography with tandem mass spectrometry;
UPCR	Urinary protein-to-creatinine.
