# Supplementary material for: Telomere Length, Oxidative Stress, and Kidney Damage Biomarkers in Fabry Nephropathy
Source: Cells. 2025 Feb 4;14(3):218. doi: 10.3390/cells14030218 (PMC11817696; doi:10.3390/cells14030218)
Supplement: Supplementary file 1 [file cells-14-00218-s001.zip › cells-3444170-supplementary.pdf]

cells

MDPI

Supplementary Materials

Telomere Length, Oxidative Stress, and Kidney Injury Biomarkers in Patients with Fabry Disease

Tina Levstek, Erazem Bahčič, Bojan Vujkovac, Andreja Cokan Vujkovac, Tine Tesovnik, Žiga Iztok Remec, Vanja Čuk and Katarina Trebušak Podkrajšek

Table S1. Variants in the *GLA* gene (reference sequence NM_000169.2) and characteristics of the included Fabry patients.

Patient	Genetic variant		Sex	Rate of progression
1	p.Arg363Pro	c.1088G>C	F	progressive nephropathy
2	p.Arg363Pro	c.1088G>C	F	progressive nephropathy
3	p.Asn272Ser	c.815A>G	F	stable kidney function
4	p.Asn272Ser	c.815A>G	M	stable kidney function
5	p.Asn272Ser	c.815A>G	F	stable kidney function
6	p.Ile270Met	c.810T>G	F	stable kidney function
7	p.Asn272Ser	c.815A>G	M	stable kidney function
8	p.Leu180Phe	c.540G>C	F	progressive nephropathy
9	p.Leu180Phe	c.540G>C	F	stable kidney function
10	p.358delGlu	c.1072_1074delGAG	M	progressive nephropathy
11	p.Arg227Ter	c.679C>T	M	progressive nephropathy
12	p.Asn272Ser	c.815A>G	F	stable kidney function
13	p.Gly261ValfsTer8	c.782delG	F	stable kidney function
14	p.Gly261ValfsTer8	c.782delG	M	stable kidney function
15	p.Ile270Met	c.810T>G	M	stable kidney function
16	p.Asn272Ser	c.815A>G	F	stable kidney function
17	p.Asn272Ser	c.815A>G	F	stable kidney function
18	p.Asn272Ser	c.815A>G	F	progressive nephropathy
19	p.Arg342Gln	c.1025G>A	M	progressive nephropathy
20	p.Glu87Asp	c.261_278del18	F	stable kidney function
21	p.Asn272Ser	c.815A>G	F	stable kidney function
22	p.Asn272Ser	c.815A>G	F	stable kidney function
23	p.Arg363Pro	c.1088G>C	M	stable kidney function
24	p.Glu87Asp	c.261_278del18	F	stable kidney function
25	p.Arg49Pro	c.146G>C	F	stable kidney function
26	p.Arg49Pro	c.146G>C	M	stable kidney function
27	p.Arg342Gln	c.1025G>A	F	stable kidney function
28	p.Arg363Pro	c.1088G>C	F	progressive nephropathy
29	p.Arg363Pro	c.1088G>C	F	stable kidney function
30	p.Asn272Ser	c.815A>G	F	stable kidney function
31	p.Arg227Ter	c.679C>T	F	stable kidney function
32	p.Cys63Tyr	c.188G>C	F	stable kidney function
33	p.Asn272Ser	c.815A>G	F	stable kidney function
34	p.Asn272Ser	c.815A>G	F	stable kidney function
35	p.Arg227Ter	c.679C>T	F	stable kidney function

Table S2. UHPLC flow profile and mobile phases ratios.

Time (min)	Flow (mL/min)	% A	% B	Curve
0	0.300	80	20	initial
0.10	0.300	80	20	6
0.65	0.300	55	45	6
5.10	0.300	45	55	1
5.60	0.300	0	100	6
8.00	0.300	0	100	6
8.50	0.300	80	20	3
10.00	0.300	80	20	1

Table S3. Comparison of urine kidney damage biomarkers between patients with stable kidney functions and those with progressive nephropathy adjusted for disease-specific therapy.

Urine marker	FD-SKF (n = 27)	FD-PN (n = 8)	FDR
IGFBP7 (mg/mol)	53.05 (30.76–74.28)	25.18 (11.39–37.60)	0.224
B2MG (mg/mol)	6.56 (4.40–12.51)	7.09 (5.46–43.59)	0.134
EGF (mg/mol)	3.91 (2.73–4.71)	1.98 (1.26–2.32)	0.027
albumin (g/mol)	1.1 (0.6–2.1)	15.1 (1.3–31.6)	0.007
OPN (mg/mol)	7.78 (4.61–11.13)	2.35 (0.46–5.40)	0.224
TFF3 (mg/mol)	4.84 (2.78–11.40)	3.23 (2.22–14.13)	0.619
UMOD (g/mol)	1.7 (0.8–3.4)	0.9 (0.2–2.2)	0.419

Table S4. Comparison of plasma kidney damage biomarkers between patients with stable kidney functions and those with progressive nephropathy adjusted for disease-specific therapy.

Plasma marker	FD-SKF (n = 27)	FD-PN (n = 8)	FDR
IGFBP7 (ng/mL)	113.5 (105.6–134.9)	156.9 (128.8–200.5)	0.105
NGAL (ng/mL)	177.2 (151.0–234.0)	267.6 (200.7–390.4)	0.017
cystatin C (μg/mL)	0.63 (0.49–0.70)	0.99 (0.86–1.51)	0.017
TIMP2 (ng/mL)	42.1 (21.6–51.5)	41.9 (20.1–60.0)	0.716
OPN (ng/mL)	4.71 (2.88–6.90)	5.42 (2.87–9.69)	0.105
TFF3 (ng/mL)	3.99 (3.21–5.92)	8.37 (6.01–11.72)	0.189
UMOD (ng/mL)	105.8 (79.0–138.9)	35.2 (21.9–46.3)	0.230
